# Evaluation of two rapid phenotypical tests—Alifax rapid AST colistin test and Rapid Polymyxin NP test—for detection of colistin resistance in *Enterobacterales*

**DOI:** 10.1007/s10096-021-04182-w

**Published:** 2021-02-17

**Authors:** Julija Germ, Katja Seme, Tjasa Cerar, Veronika Krizan Hergouth, Mateja Pirs

**Affiliations:** 1 Institute of Microbiology and Immunology, Faculty of Medicine, University of Ljubljana, Ljubljana, Slovenia; 2 Diagnostic & Research Institute of Hygiene, Microbiology and Environmental Medicine, Medical University of Graz, Graz, Austria

**Keywords:** Colistin resistance, Rapid diagnostic test, Enterobacterales, susceptibility testing

## Abstract

Our study evaluates the performance of two rapid phenotypical tests to detect colistin resistance in *Enterobacterales*: Alifax rapid AST colistin test using the HB&L system and Rapid Polymyxin NP test prepared in-house. A collection of well-characterized 53 colistin-susceptible and 66 colistin-resistant*Enterobacterales* isolates was used. The results obtained using both rapid tests were compared to the reference broth microdilution. Overall categorical agreement was 81.5% for Alifax test and 98.3% for Rapid Polymyxin NP test. Based on our results, the Rapid Polymyxin NP test is superior to the Alifax test that performed inadequate for *Enterobacter* spp.

**Supplementary Information:**

The online version contains supplementary material available at 10.1007/s10096-021-04182-w.

## Introduction

Colistin use in clinical practice is mainly restricted to treatment of severe infections caused by multidrug-resistant (MDR) Gram-negative bacilli (GNB) [1]. Hence, it is rarely included in routine susceptibility panels in medical microbiology laboratories. In addition, colistin susceptibility testing is methodologically challenging due to its inherent properties [1–3]. The reference method chosen by the CLSI-EUCAST joint subcommittee is broth microdilution (BMD) [4]. Because colistin susceptibility testing is usually performed on demand when it becomes a treatment option results are delayed for up to 24 h. To reduce the time to results and provide simpler testing, several rapid tests have been developed [5–7].

This study evaluates the performance of two rapid tests that detect colistin resistance in *Enterobacterales* isolates and compares the results with the reference BMD.

The Biochemical Rapid Polymyxin NP test, developed by Nordmann et al., is a well-studied rapid test that detects bacterial growth in the presence of a defined concentration of colistin that is near the EUCAST breakpoint [5, 8–10].

The HB&L system (Alifax, Polverara, Italy) for bacterial growth detection and antimicrobial susceptibility testing is an automated liquid-culture system established on a proprietary light-scattering based technology. The system detects growth of bacteria through monitoring the broth turbidity level using a McFarland monitor. It allows antimicrobial susceptibility testing based on a modified MIC method, with an antimicrobial concentration near the EUCAST breakpoint. Susceptibility results are obtained within 5 h. The rapid AST colistin *Enterobacteriaceae*/EU (Alifax) test (hereinafter: the Alifax rapid COL-AST test) using the HB&L system was evaluated in this study [11–13].

## Methods

To evaluate the two rapid tests, a total of 119 well-characterized *Enterobacterales* isolates were used; the majority were collected prospectively at the Institute of Microbiology and Immunology, Faculty of Medicine, University of Ljubljana between February 2017 and June 2018 [14, 15]. Isolates were recovered in accordance with our standard laboratory protocol and identified using matrix-assisted laser desorption/ionization time-of-flight mass-spectrometry (MALDI TOF MS) (Microflex LT, Bruker Daltonics, Bremen, Germany). Isolates were stored at − 80 °C. Non-selective culture medium Columbia agar plate (Oxoid, Vienna, Austria) was used for reviving.

Colistin MIC was determined in accordance with the joint CLSI-EUCAST Polymyxin Breakpoints Working Group recommendation. MIC results were interpreted in accordance with EUCAST guidelines [16].

Our collection consisted of 53 colistin-susceptible isolates and 66 isolates with acquired colistin resistance. The colistin-resistant strains were 29 *Escherichia coli*, of which 15 were MCR-producers from an international collection (14 *mcr-1* and a single *mcr-2* isolate), 16 *Klebsiella pneumoniae*, 19 *Enterobacter* spp., and two *Citrobacter* spp. (Appendix) [17–20].

To perform *mcr* gene detection, genomic DNA was isolated using Instant Gene Matrix (Bio-Rad Laboratories, Hercules, USA) following the manufacturer’s instructions. Detection of *mcr-1*, *mcr-2*, *mcr-3*, *mcr-4*, and *mcr-5* was performed on isolates with colistin MIC > 2 mg/l using multiplex PCR as previously described [14, 15, 21].

To perform the susceptibility testing of *Enterobacterales* isolates using the Alifax rapid COL-AST test, a 10-μl loopful of fresh overnight bacterial culture was carried out by transferring from non-selective culture medium Columbia agar plate (Oxoid) into a vial containing 3 ml of HB&L culture kit (Alifax) enrichment broth. Vials were loaded into the HB&L system (Alifax) and bacterial growth was automatically monitored; a notification on the screen appeared when 0.5 McFarland optical density was reached. Meanwhile, lyophilized colistin powder (Alifax) was dissolved in 2 ml of regenerating solution. Two vials containing enrichment broth were prepared for each bacterial isolate following the manufacturer’s instructions; into the vial for susceptibility testing, 200 μl of colistin suspension was added; the other vial served as a reference. Into each of the two vials, 100 μl of 0.5 McFarland of the bacterial suspension was transferred. The vials were inserted into the HB&L system (Alifax) following the manufacturer’s protocol for 5 h. Bacterial growth was automatically monitored while the logarithm of growth was calculated and compared between the two vials. The final results for the bacterial isolate were reported by the HB&L system as colistin-susceptible and colistin-resistant using EUCAST breakpoints (2 mg/l for *Enterobacterales*).

The Rapid Polymyxin NP Test was prepared in-house as previously described [5]. The trays were visually inspected after 10 min, followed by hourly inspection after 1 h, 2 h, 3 h, and 4 h for color change.

The results obtained with both rapid methods were compared to the reference BMD method and categorized as follows: categorical agreement (CA) between the rapid test and the reference method, major error (ME, defined as false-resistant compared to the reference BMD method), and very major error (VME, defined as false-susceptible compared to the reference BMD method), as described elsewhere [22, 23]. Positive predictive value (PPV), negative predictive value (NPV) sensitivity, and specificity were calculated.

## Results

Using the Alifax rapid COL-AST test, CA was attained in 97/119 isolates (81.5%). Among the colistin-susceptible isolates CA was 100%, and among the 66 colistin-resistant isolates CA attained 66.7% (44/66 isolates). CA determined per species/genus was 97.8% for *Escherichia coli*, 97.8% for *K. pneumoniae*, 51.6% for *Enterobacter* spp., and 100.0% for *Citrobacter* spp. Overall VME was detected in 22 out of 119 *Enterobacteriaceae*: 1/46 for *E. coli*, 6/32 for *K. pneumoniae*, and 15/31 for *Enterobacter aerogenes*. Detailed results are shown in Table 1.

**Table 1:** Performance of the two rapid tests for detection of colistin resistance compared to the reference broth microdilution method using 119 *Enterobacterales* isolates

(*n*, colistin MIC range in mg/l)	Bacteria tested				
	*Enterobacterales* (*n* 119, 0.25–128)	*Escherichia coli* (*n* 46, 0.25–8)	*Klebsiella pneumoniae* (*n* 38, 0.25–64)	*Citrobacter* spp. (*n* 4, 0.25–64)	*Enterobacter* spp. (*n* 31, 0.25–128)
Alifax rapid AST colistin test (Alifax, Polverara, Italy)					
CA (*n*)	97 (81.5%)	45 (97.8%)	32 (84.2%)	4 (100%)	16 (51.6%)
ME (*n*)	0	0	0	0	0
VME (*n*)	22 (18.5%)	1 (2.2%)	6 (15.8%)	0	15 (48.4%)
PPV (%)	100	100	100	100	100
NPV (%)	71.1	94.7	78.6	100	44.4
Sensitivity (%)	66	96	63	100	21
Specificity (%)	100	100	100	100	100
Rapid Polymyxin NP test					
CA (*n*)	117 (98.3%)	46 (100%)	38 (100%)	4 (100%)	29 (93.5%)
ME (*n*)	0	0	0	0	0
VME (*n*)	2 (1.7%)	0	0	0	2 (6.5%)
PPV (%)	100	100	100	100	100
NPV (%)	96.4	100	100	100	85.7
Sensitivity (%)	97	100	100	100	89
Specificity (%)	100	100	100	100	100

*N* number, *MIC* minimal inhibitory concentration, *AST* antimicrobial susceptibility testing, *CA* categorical agreement, *ME* major error, *VME* very major error, *PPV* positive predictive value, *NPV* negative predictive value

Using the in-house Rapid Polymyxin NP test, CA was attained in 117/119 isolates (98.3%). Among the colistin-susceptible isolates, CA was 100%. Among the 66 colistin-resistant isolates, CA determined per species/genus was 100.0% for *E. coli*, 100.0% for *K. pneumoniae*, 88.9% for *Enterobacter* spp., and 100.0% for *Citrobacter* spp. In total, VME was detected in 2/119 *Enterobacterales*; both were *Enterobacter* spp. Detailed results are shown in Table 1.

## Discussion

A total of 119 isolates were included in this evaluation of two rapid phenotypical tests to detect colistin resistance in *Enterobacterales*. 

The performance of the Rapid Polymyxin NP test was excellent, with overall 98.3% CA and 2.17% VME (two colistin-resistant *Enterobacter* spp. isolates with colistin MICs of 32 and 128 mg/l tested false-colistin-susceptible using this test), which is in accordance with previous evaluations [5, 8–10].

The HB&L system yielded an 81.5% overall CA and no ME; however, 18.5% of the evaluated isolates tested as false-colistin-susceptible (VME), including one *E. coli* (not an MCR producer), six *K. pneumoniae*, and 15 of 31 *Enterobacter* spp. isolates. Although the test performed well for *E. coli* and to a lesser extent for *K. pneumoniae*, the performance of susceptibility testing for *Enterobacter* spp. was inadequate. We have noted that growth of colistin-resistant *Enterobacter* spp. isolates was actually present in the vials upon visual inspection after the test was completed (turbid broth); however, the system algorithm failed to detect resistance (no growth present was reported). Perhaps with an improved algorithm the performance of the HB&L system for *Enterobacter* spp. will improve. Another possibility for such a discrepancy could be that *Enterobacter* spp. testing requires a longer time. However, the Rapid Polymyxin NP test performed much better with the same isolates of this challenging genus even with a shorter test time (Alifax 5 h, Rapid Polymyxin NP 2 h), and so this explanation is unlikely [24]. Of note is also the previously described trend toward forming a heteroresistant subpopulation for colistin in *Enterobacter* spp.; the smaller bacterial inoculum used for the HB&L system (Alifax) compared to the one used in the Rapid Polymyxin NP test could potentially result in a smaller number of resistant bacterial cells. In combination with a shorter incubation time in comparison with standard MIC testing, this may lead to false-susceptibility results. Interestingly, false-negative results were described among *Enterobacter* spp. when evaluating the Rapid Polymyxin NP Test [10]. Among the 31 *Enterobacter* spp. isolates included in our performance evaluation, 22 were *Enterobacter aerogenes* and nine isolates belonged to the *Enterobacter cloacae* complex. In a study conducted by Simar et al., a higher number of *Enterobacter* spp. isolates were included in the evaluation with a different proportion of *E. cloacae* and *E. aerogenes* (in favor of *E. aerogenes*), which could be an explanation for the difference in the results [10].

Based on our experience, the Alifax rapid COL-AST test requires longer hands-on time compared to the Rapid Polymyxin NP test due to its two-step course.

Interpretation of the results is automated in the HB&L system (Alifax); in contrast, the Rapid Polymyxin NP test results are based on color change and are visually inspected, which can lead to subjective interpretation. In our test, the color change was well-pronounced and interpretation after 2 h was not problematic. Furthermore, readout optimization with an ELISA reader has been described, which enhances the objectivity of the results [25].

To conclude, the use of rapid tests to determine colistin resistance is a promising tool to help assess the suitability of using colistin when MDRGNB are isolated in patients with severe infection. The result for colistin can realistically be obtained the same working day the initial antimicrobial susceptibility is available. Based on the results of our study, the Rapid Polymyxin NP test is superior to the Alifax rapid COL-AST test that performed inadequate in detection of colistin-resistant *Enterobacter* spp.

## Supplementary Information

ESM 1 (DOCX 130 kb)

## Data Availability

The data that support the findings of this study are available from the corresponding author upon reasonable request.
