# Supplementary material for: Evaluation of two rapid phenotypical tests—Alifax rapid AST colistin test and Rapid Polymyxin NP test—for detection of colistin resistance in Enterobacterales
Source: Eur J Clin Microbiol Infect Dis. 2021 Feb 17;40(8):1749–53. doi: 10.1007/s10096-021-04182-w (PMC8295142; doi:10.1007/s10096-021-04182-w)
Supplement: Supplementary file 1 — (DOCX 130 kb) [file 10096_2021_4182_MOESM1_ESM.docx]

**Technical appendix:** The study was performed using a collection of 119 *Enterobacterales* isolates which 13 *mcr-1–*positive *E. coli* strains from four laboratories, kindly provided by Laurent Poirel and Patrice Nordmann (five strains), Vittorio Sambri (five strains), and the Austrian National Reference Centre for Nosocomial Infections and Antimicrobial Resistance (two strains), plus a single Slovenian *mcr-1*–positive *E. coli*, as well as an NCTC 13864 *mcr-1*–positive *E. coli*. An *mcr-2* positive *E. coli* was kindly provided by the University of Antwerp, Belgium.

Colistin minimal inhibitory concentrations, determined by the reference broth microdilution and the two rapid tests for detection of colistin resistance were performed from the same non-selective culture medium. The results are listed below.

|  | **Bacterial isolate** | ***mcr(1-5)* genes presence** | **Colistin MIC by reference BMD (mg/L)** | **Alifax rapid AST colistin test (Alifax, Polverara Italy)**  **result** | | | **Rapid Polymyxin NP test result** | |
| --- | --- | --- | --- | --- | --- | --- | --- | --- |
| **Colistin resistant *Enterobacterales* isolates** | | | | | | | | |
| 1 | *Escherichia coli* HK 11/21 | ND | 4 | TP | CA | TP | | CA |
| 2 | *Escherichia coli* HK 6/32 | ND | 4 | TP | CA | TP | | CA |
| 3 | *Escherichia coli* 132/6 | ND | 4 | **FN** | **VME** | TP | | CA |
| 4 | *Escherichia coli* 74/2 | ND | 8 | TP | CA | TP | | CA |
| 5 | *Escherichia coli* 186/9 | ND | 4 | TP | CA | TP | | CA |
| 6 | *Escherichia coli* 245/8 | ND | 4 | TP | CA | TP | | CA |
| 7 | *Escherichia coli* 42/12 | ND | 8 | TP | CA | TP | | CA |
| 8 | *Escherichia coli* 367/1 | ND | 4 | TP | CA | TP | | CA |
| 9 | *Escherichia coli* 328/3 | ND | 4 | TP | CA | TP | | CA |
| 10 | *Escherichia coli* 284/11 | *mcr-1* | 4 | TP | CA | TP | | CA |
| 11 | *Escherichia coli* 191/10 | ND | 8 | TP | CA | TP | | CA |
| 12 | *Escherichia coli* 442/10 | ND | 4 | TP | CA | TP | | CA |
| 13 | *Escherichia coli* TJ | ND | 8 | TP | CA | TP | | CA |
| 14 | *Escherichia coli* KJ | ND | 4 | TP | CA | TP | | CA |
| 15 | *Escherichia coli* ME | ND | 4 | TP | CA | TP | | CA |
| 16 | *Escherichia coli* MCR-1 NCTC 13846 | *mcr-1* | 4 | TP | CA | TP | | CA |
| 17 | *Escherichia coli* MCR-2 Belgium | *mcr-2* | 4 | TP | CA | TP | | CA |
| 18 | *Escherichia coli* AUT 1 | *mcr-1* | 4 | TP | CA | TP | | CA |
| 19 | *Escherichia coli* AUT 2 | *mcr-1* | 4 | TP | CA | TP | | CA |
| 20 | *Escherichia coli* NP 1 | *mcr-1* | 4 | TP | CA | TP | | CA |
| 21 | *Escherichia coli* NP 2 | *mcr-1* | 4 | TP | CA | TP | | CA |
| 22 | *Escherichia coli* NP 3 | *mcr-1* | 4 | TP | CA | TP | | CA |
| 23 | *Escherichia coli* NP 4 | *mcr-1* | 4 | TP | CA | TP | | CA |
| 24 | *Escherichia coli* NP 5 | *mcr-1* | 4 | TP | CA | TP | | CA |
| 25 | *Escherichia coli* IT 1 | *mcr-1* | 8 | TP | CA | TP | | CA |
| 26 | *Escherichia coli* IT 2 | *mcr-1* | 8 | TP | CA | TP | | CA |
| 27 | *Escherichia coli* IT 3 | *mcr-1* | 8 | TP | CA | TP | | CA |
| 28 | *Escherichia coli* IT 4 | *mcr-1* | 8 | TP | CA | TP | | CA |
| 29 | *Escherichia coli* IT 5 | *mcr-1* | 8 | TP | CA | TP | | CA |
| 30 | *Klebsiella pneumoniae* 137/2 | ND | 16 | TP | CA | TP | | CA |
| 31 | *Klebsiella pneumoniae* 64/12 | ND | 16 | TP | CA | TP | | CA |
| 32 | *Klebsiella pneumoniae* 61/1 | ND | 32 | **FN** | **VME** | TP | | CA |
| 33 | *Klebsiella pneumoniae* 84/8 | ND | 64 | **FN** | **VME** | TP | | CA |
| 34 | *Klebsiella pneumoniae* 41/3 | ND | 16 | **FN** | **VME** | TP | | CA |
| 35 | *Klebsiella pneumoniae* 69/12 | ND | 8 | TP | CA | TP | | CA |
| 36 | *Klebsiella pneumoniae* 26/2 | ND | 4 | TP | CA | TP | | CA |
| 37 | *Klebsiella pneumoniae* 226/2 | ND | 16 | TP | CA | TP | | CA |
| 38 | *Klebsiella pneumoniae* 229/6 | ND | 4 | TP | CA | TP | | CA |
| 39 | *Klebsiella pneumoniae* 158/11 | ND |  | TP | CA | TP | | CA |
| 40 | *Klebsiella pneumoniae* 46/10 | ND | 16 | **FN** | **VME** | TP | | CA |
| 41 | *Klebsiella pneumoniae* 43/3 | ND | 4 | **FN** | **VME** | TP | | CA |
| 42 | *Klebsiella pneumoniae* 39/5 | ND | 4 | TP | CA | TP | | CA |
| 43 | *Klebsiella pneumoniae* 275/1 | ND | 32 | **FN** | **VME** | TP | | CA |
| 44 | *Klebsiella pneumoniae* 219/5 | ND | 16 | TP | CA | TP | | CA |
| 45 | *Klebsiella pneumoniae* TU | ND | 4 | TP | CA | TP | | CA |
| 46 | *Enterobacter* spp. GL | ND | 8 | **FN** | **VME** | TP | | CA |
| 47 | *Enterobacter* spp. 301/8 | ND | 16 | TP | CA | TP | | CA |
| 48 | *Enterobacte*r spp. 153/4 | ND | 32 | **FN** | **VME** | TP | | CA |
| 49 | *Enterobacter* spp. 498/8 | ND | 32 | TP | CA | **FN** | | **VME** |
| 50 | *Enterobacter* spp.305/2 | ND | 32 | **FN** | **VME** | TP | | CA |
| 51 | *Enterobacter* spp. 81/3 | ND | 32 | **FN** | **VME** | TP | | CA |
| 52 | *Enterobacter* spp. 67/7 | ND | 16 | TP | CA | TP | | CA |
| 53 | *Enterobacter* spp. 276/3 | ND | 16 | **FN** | **VME** | TP | | CA |
| 54 | *Enterobacter* spp. 342/4 | ND | 64 | **FN** | **VME** | TP | | CA |
| 55 | *Enterobacter* spp. 41/8 | ND | 16 | TP | CA | TP | | CA |
| 56 | *Enterobacter* spp. 140/7 | ND | 8 | **FN** | **VME** | TP | | CA |
| 57 | *Enterobacter* spp. 165/3 | ND | 128 | **FN** | **VME** | TP | | CA |
| 58 | *Enterobacter* spp. 95/3 | ND | 128 | **FN** | **VME** | TP | | CA |
| 59 | *Enterobacter* spp. 104/9 | ND | 128 | **FN** | **VME** | **FN** | | **VME** |
| 60 | *Enterobacter* spp. 485/10 | ND | 64 | **FN** | **VME** | TP | | CA |
| 61 | *Enterobacter* spp. 244/1 | ND | 128 | **FN** | **VME** | TP | | CA |
| 62 | *Enterobacter* spp. 106/10 | ND | 128 | **FN** | **VME** | TP | | CA |
| 63 | *Enterobacte*r spp. 286/4 | ND | 64 | **FN** | **VME** | TP | | CA |
| 64 | *Enterobacter* spp. 94/6 | ND | 64 | **FN** | **VME** | TP | | CA |
| 65 | *Citrobacter* spp. 261/12 | ND | 64 | TP | CA | TP | | CA |
| 66 | *Citrobacter* spp. 493/5 | ND | 128 | TP | CA | TP | | CA |
| **Colistin-susceptible *Enterobacterales*isolates** | | | | | | | | |
| 67 | *Escherichia coli* 141/6 | NA | 0.5 | TN | CA | TN | | CA |
| 68 | *Escherichia coli* 88/6 | NA | 0.5 | TN | CA | TN | | CA |
| 69 | *Escherichia coli* 139/6 | NA | 0.5 | TN | CA | TN | | CA |
| 70 | *Escherichia coli* 90/10 | NA | 0.25 | TN | CA | TN | | CA |
| 71 | *Escherichia coli* 156/2 | NA | 0.5 | TN | CA | TN | | CA |
| 72 | *Escherichia coli* 129/1 | NA | 0.5 | TN | CA | TN | | CA |
| 73 | *Escherichia coli* 104/5 | NA | 0.25 | TN | CA | TN | | CA |
| 74 | *Escherichia coli* 77/11 | NA | 0.5 | TN | CA | TN | | CA |
| 75 | *Escherichia coli* 129/4 | NA | 0.5 | TN | CA | TN | | CA |
| 76 | *Escherichia coli* 57/3 | NA | 0.5 | TN | CA | TN | | CA |
| 77 | *Escherichia coli* 90/10 | NA | 1 | TN | CA | TN | | CA |
| 78 | *Escherichia coli* 104/5 | NA | 0.5 | TN | CA | TN | | CA |
| 79 | *Escherichia coli* 123/8 | NA | 0.5 | TN | CA | TN | | CA |
| 80 | *Escherichia coli* 129/4 | NA | 1 | TN | CA | TN | | CA |
| 81 | *Escherichia coli* 129/1 | NA | 1 | TN | CA | TN | | CA |
| 82 | *Escherichia coli* 138/2 | NA | 2 | TN | CA | TN | | CA |
| 83 | *Escherichia coli* ATCC 25922 | NA | 0.5 | TN | CA | TN | | CA |
| 84 | *Klebsiella pneumoniae* HK 18/20 | NA | 0.5 | TN | CA | TN | | CA |
| 85 | *Klebsiella pneumoniae* 132/9 | NA | 2 | TN | CA | TN | | CA |
| 86 | *Klebsiella pneumoniae* 156/9 | NA | 0.5 | TN | CA | TN | | CA |
| 87 | *Klebsiella pneumoniae* 158/2 | NA | 1 | TN | CA | TN | | CA |
| 88 | *Klebsiella pneumoniae* 89/7 | NA | 0.5 | TN | CA | TN | | CA |
| 89 | *Klebsiella pneumoniae* 92/9 | NA | 1 | TN | CA | TN | | CA |
| 90 | *Klebsiella pneumoniae* 147/1 | NA | 2 | TN | CA | TN | | CA |
| 91 | *Klebsiella pneumoniae* 73/7 | NA | 0.5 | TN | CA | TN | | CA |
| 92 | *Klebsiella pneumoniae* 51/1 | NA | 0.25 | TN | CA | TN | | CA |
| 93 | *Klebsiella pneumoniae* 259/1 | NA | 0.5 | TN | CA | TN | | CA |
| 94 | *Klebsiella pneumoniae* 249/7 | NA | 0.5 | TN | CA | TN | | CA |
| 95 | *Klebsiella pneumoniae* 51/2 | NA | 0.25 | TN | CA | TN | | CA |
| 96 | *Klebsiella pneumoniae* 138/7 | NA | 0.25 | TN | CA | TN | | CA |
| 97 | *Klebsiella pneumoniae* 158/5 | NA | 1 | TN | CA | TN | | CA |
| 98 | *Klebsiella pneumoniae* 136/6 | NA | 0.5 | TN | CA | TN | | CA |
| 99 | *Klebsiella pneumoniae* 88/4 | NA | 0.5 | TN | CA | TN | | CA |
| 100 | *Klebsiella pneumoniae* 89/7 | NA | 0.5 | TN | CA | TN | | CA |
| 101 | *Klebsiella pneumoniae* 89/4 | NA | 1 | TN | CA | TN | | CA |
| 102 | *Klebsiella pneumoniae* 92/4 | NA | 1 | TN | CA | TN | | CA |
| 103 | *Klebsiella pneumoniae* 92/1 | NA | 0.5 | TN | CA | TN | | CA |
| 104 | *Klebsiella pneumoniae* 92/9 | NA | 1 | TN | CA | TN | | CA |
| 105 | *Klebsiella pneumoniae* 129/2 | NA | 2 | TN | CA | TN | | CA |
| 106 | *Enterobacter* spp. 85/6 | NA | 0.5 | TN | CA | TN | | CA |
| 107 | *Enterobacter* spp. 278/12 | NA | 0.5 | TN | CA | TN | | CA |
| 108 | *Enterobacter* spp. 206/8 | NA | 0.5 | TN | CA | TN | | CA |
| 109 | *Enterobacter* spp. 251/5 | NA | 0.5 | TN | CA | TN | | CA |
| 110 | *Enterobacter* spp. 61/2 | NA | 0.5 | TN | CA | TN | | CA |
| 111 | *Enterobacter* spp. 56/10 | NA | 0.5 | TN | CA | TN | | CA |
| 112 | *Enterobacter* spp. 56/3 | NA | 0.25 | TN | CA | TN | | CA |
| 113 | *Enterobacter* spp. 73/6 | NA | 0.5 | TN | CA | TN | | CA |
| 114 | *Enterobacter* spp. 87/2 | NA | 0.5 | TN | CA | TN | | CA |
| 115 | *Enterobacter* spp. 90/9 | NA | 1 | TN | CA | TN | | CA |
| 116 | *Enterobacter* spp. 123/6 | NA | 0.5 | TN | CA | TN | | CA |
| 117 | *Enterobacter* spp. 172/8 | NA | 0.5 | TN | CA | TN | | CA |
| 118 | *Citrobacter* spp 56/6 | NA | 0.5 | TN | CA | TN | | CA |
| 119 | *Citrobacter* spp 125/3 | NA | 0.5 | TN | CA | TN | | CA |
| MIC minimal inhibitory concentration, BMD broth microdilution, ND not detected, TP true positive, CA categorical agreement, FN false negative, VME very major error, TN true negative, NA not applicable | | | | | | | | |
